# Non-contrast-enhanced CT texture analysis of primary and metastatic pancreatic ductal adenocarcinomas: value in assessment of histopathological grade and differences between primary and metastatic lesions

**DOI:** 10.1007/s00261-022-03646-7

**Published:** 2022-09-14

**Authors:** Michael Janisch, Gabriel Adelsmayr, Heimo Müller, Andreas Holzinger, Elmar Janek, Emina Talakic, Michael Fuchsjäger, Helmut Schöllnast

**Affiliations:** 1 Division of General Radiology, Department of Radiology, Medical University of Graz, Graz, Austria; 2 Diagnostic and Research Center for Molecular BioMedicine, Diagnostic and Research Institute of Pathology, Medical University of Graz, Graz, Austria; 3 Institute for Medical Informatics, Statistics and Documentation, Medical University of Graz, Graz, Austria; 4 Institute of Radiology, LKH Graz II, Graz, Austria

**Keywords:** Tomography, X-ray computed, Pancreas cancer, Histopathology, Neoplasm metastases

## Abstract

**Purpose:**

To evaluate the utility of non-contrast-enhanced CT texture analysis (CTTA) for predicting the histopathological differentiation of pancreatic ductal adenocarcinomas (PDAC) and to compare non-contrast-enhanced CTTA texture features between primary PDAC and hepatic metastases of PDAC.

**Methods:**

This retrospective study included 120 patients with histopathologically confirmed PDAC. Sixty-five patients underwent CT-guided biopsy of primary PDAC, while 55 patients underwent CT-guided biopsy of hepatic PDAC metastasis. All lesions were segmented in non-contrast-enhanced CT scans for CTTA based on histogram analysis, co-occurrence matrix, and run-length matrix. Statistical analysis was conducted for 372 texture features using Mann–Whitney *U* test, Bonferroni–Holm correction, and receiver operating characteristic (ROC) analysis. A *p* value < 0.05 was considered statistically significant.

**Results:**

Three features were identified that differed significantly between histopathological G2 and G3 primary tumors. Of these, “low gray-level zone emphasis” yielded the largest AUC (0.87 ± 0.04), reaching a sensitivity and specificity of 0.76 and 0.83, respectively, when a cut-off value of 0.482 was applied. Fifty-four features differed significantly between primary and hepatic metastatic PDAC.

**Conclusion:**

Non-contrast-enhanced CTTA of PDAC identified differences in texture features between primary G2 and G3 tumors that could be used for non-invasive tumor assessment. Extensive differences between the features of primary and metastatic PDAC on CTTA suggest differences in tumor microenvironment.

**Graphical Abstract:**

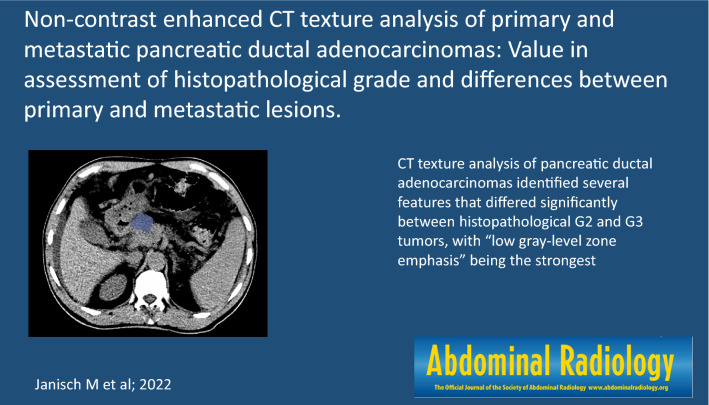

## Introduction

According to recent data, pancreatic ductal adenocarcinoma (PDAC) is still the seventh leading cause of global cancer deaths, mainly because patients remain free of symptoms for a long time and the diagnosis is often made at an advanced stage. The poor prognosis for PDAC is further worsened by the presence of distant metastases, which are found in more than 50% of patients at the time of diagnosis, most commonly in the liver [1].

Among local and distant extension, the histopathological grade of differentiation of PDAC, which is described by tumor architecture, cell morphology, mitotic activity, and cell nucleus polymorphisms, serves a major prognostic factor [2]. While well-differentiated G1 tumors are predominantly arranged in tubular structures and show low mitotic activity and a low number of polymorphisms, poorly differentiated G3 tumors are mainly configured in solid areas and have high mitotic activity and a high number of polymorphisms [3]. Poorly differentiated PDAC is more aggressive and associated with shorter survival than well-differentiated PDAC, and the risks associated with surgical resection may outweigh the benefits of the procedure [4, 5]. In addition, neoadjuvant therapy may improve survival in patients with poorly differentiated PDAC [6]. To estimate patients’ survival and adapt treatment, a non-invasive technique for tumor assessment is of common interest. Previous studies have shown that tumor heterogeneity is an important biomarker for patient survival [7, 8]. It is determined by, among other factors, cellular density, angiogenesis, and the presence of micronecrosis and can be quantified noninvasively using CT texture analysis (CTTA) [9–11]. A statistically significant correlation has been found between CTTA of locally advanced, non-metastatic PDAC, and overall survival [12]. A recent study showed for the first time that the histopathological grade of PDAC could be discriminated using texture analysis and machine learning [13]. In that study, contrast-enhanced CT was utilized with non-uniform CT scan parameters [13]. However, it has been reported that contrast media in CTTA may be a source of error due to variability among factors, such as contrast medium volume, concentration, and injection rate, as well as patient’s allometry and cardiac output [14, 15]. Therefore, the primary aim of our study was to assess whether non-enhanced CTTA of PDAC allows prediction of the histopathological grade of differentiation. In addition, we aimed to assess whether differences could be found on non-enhanced CTTA between primary and metastatic PDAC, which could reflect different histopathological characteristics.

## Methods

### Patient population

**Fig. 1:**
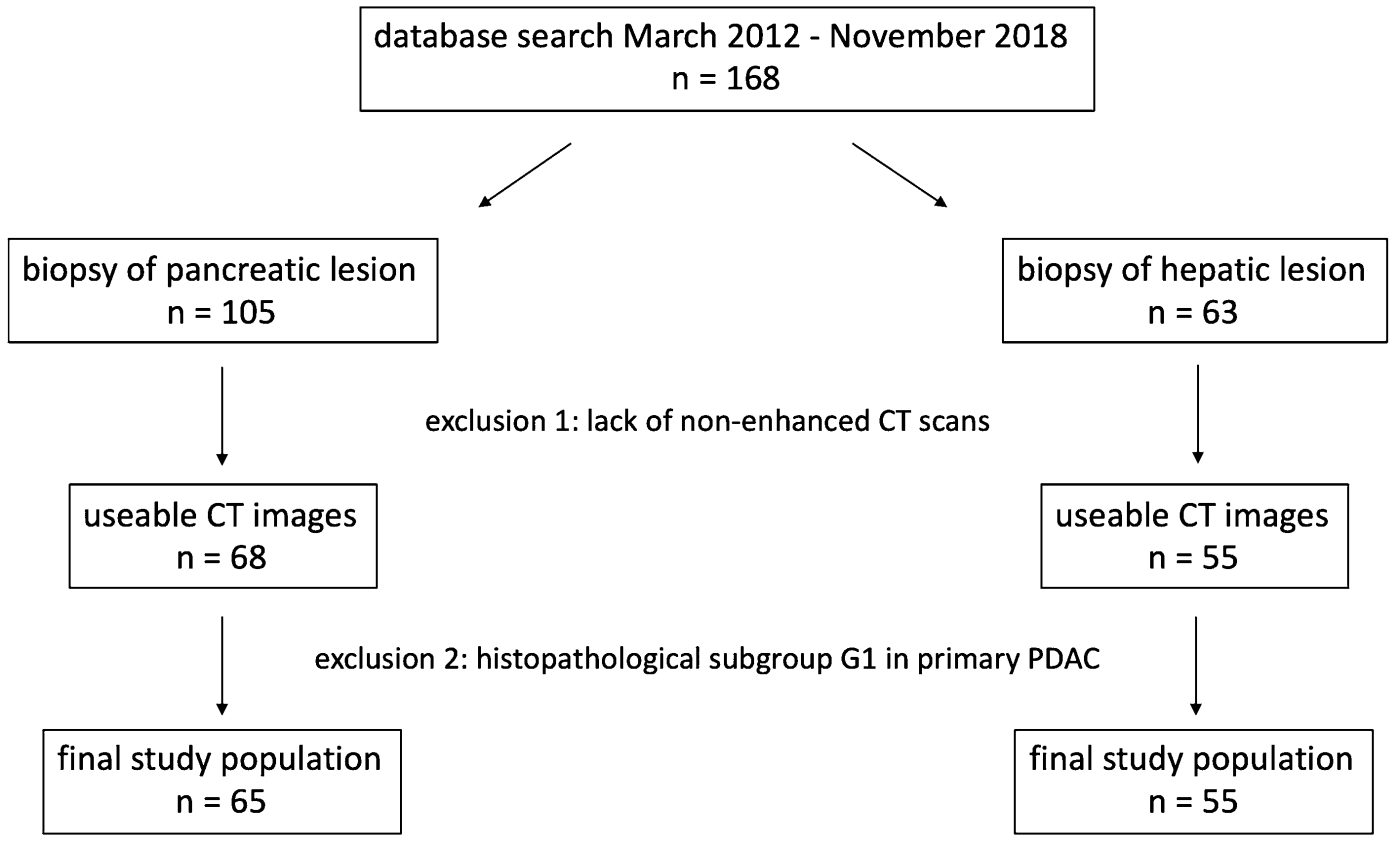
Flowchart of the study population

Our institutional review board approved this retrospective study and waived the requirement for written informed consent. A computer database search for the period from March 2012 to November 2018 yielded 105 consecutive patients, who underwent CT-guided biopsy of a pancreatic tumor at our institution and received a histopathological diagnosis of PDAC. Moreover 63 additional patients with a pancreatic tumor and suspicious liver lesions underwent CT-guided biopsy of the liver, resulting in a histopathological diagnosis of adenocarcinoma consistent with PDAC metastasis were included in our study. The indication for biopsy in the former group of patients was previous imaging findings suspicious for locally advanced PDAC, while in the latter group of patients, it was previous imaging findings suspicious for metastatic PDAC. Out of a total of 168 patients, 45 were excluded due to lack of a non-enhanced scan from the CT-guided biopsy procedure. In addition, because only 3 patients had primary PDAC of histopathological G1, a valid analysis of this subgroup was impossible, and these patients had to be excluded as well. Thus, the final study population consisted of 65 patients (28 females, 37 males) who underwent CT-guided biopsy of the pancreas with histopathologically confirmed PDAC and 55 patients (22 females, 33 males) who underwent CT-guided biopsy of the liver with histopathologically confirmed PDAC metastasis. According to the medical records, none of the patients received anti-cancer therapy before biopsy. Figure 1 shows a flowchart of the study population selection process.

At biopsy out of the 65 patients with primary PDAC alone, 23 patients had histopathological G2 and 42 had histopathological G3 tumors.

Patients’ demographic details and tumor characteristics are summarized in Table 1.

**Table 1:** Patients’ demographic, histopathological tumor grade, and tumor size data (*n* = 120)

Tumor group	Number of patients	Mean age ± SD (range) in years	Gender (f/m)	Tumor size (range) in mm
Primary PDAC	65	67 ± 9 (41–86)	28/37	35 ± 12 (19–89)
G2	23	67 ± 7.5 (48–77)	13/10	32 ± 11 (19–76)
G3	42	67 ± 10 (41–86)	15/27	39 ± 14 (22–89)
Hepatic metastasis	55	66 ± 11 (38–88)	22/33	31 ± 18 (11–87)

*SD* standard deviation, *PDAC* pancreatic adenocarcinoma

### CT imaging

CT imaging was performed as part of CT-guided biopsy of the pancreas or the liver. All CT-guided biopsies were performed on a single-multislice CT scanner (Somatom Definition AS +, Siemens Healthineers). Pre-interventional non-enhanced CT scans were performed to localize the mass and plan the upcoming procedure using scanning parameters as follows: tube voltage, 120 kV; tube current, 100 mAs with tube current modulation; collimation, 128 × 0.6 mm; pitch, 1.2; reconstruction thickness, 5 mm; reconstruction kernel and standard kernel using iterative reconstruction; and increment, 3.0 mm.

### Texture analysis

**Fig. 2:**
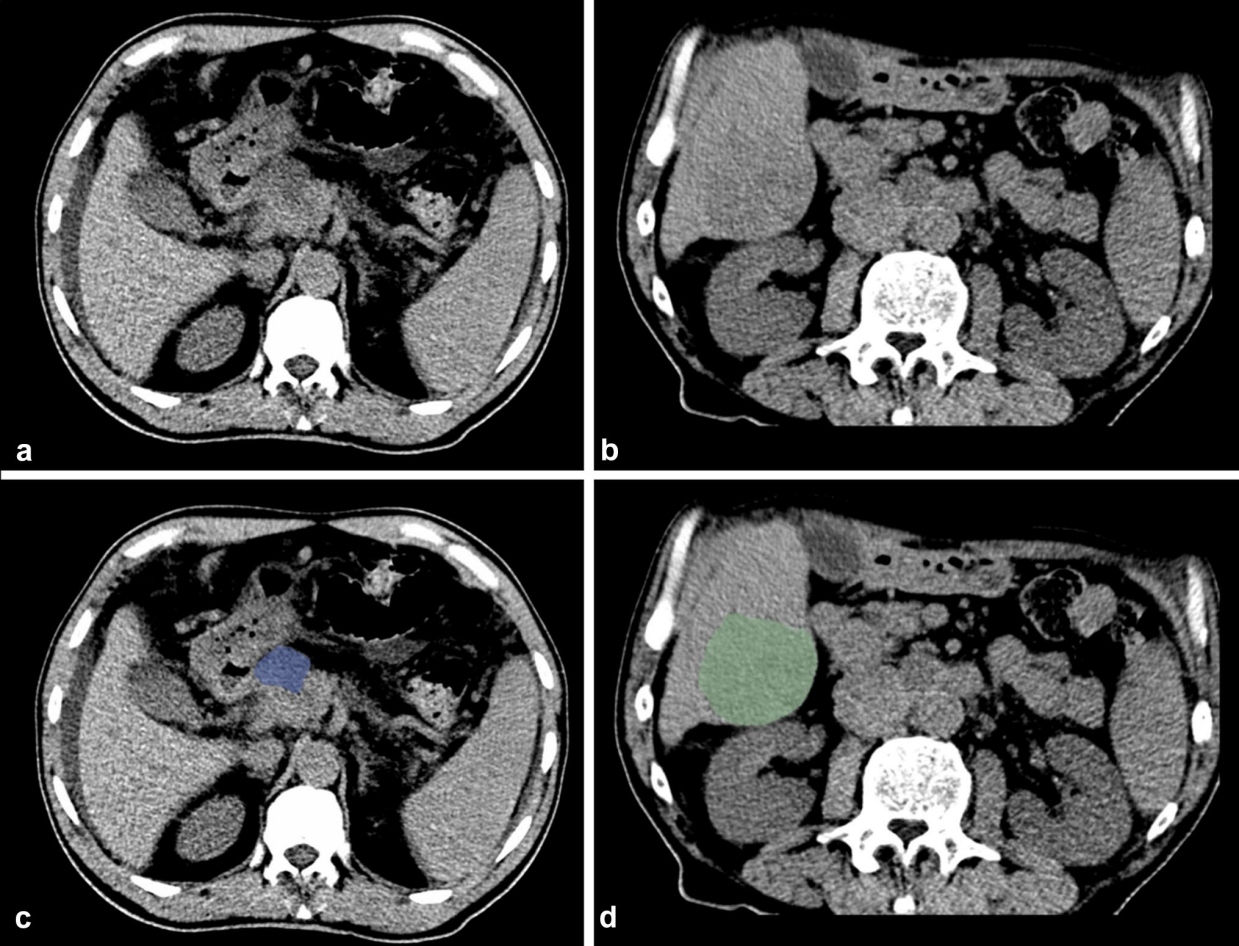
**a–d** 2D segmentation of primary PDAC (**a**, **c**) and hepatic metastasis of PDAC (**b**, **d**) on axial, non-enhanced CT. To prevent inclusion of any adjacent vessels or tissue, a small distance from the edge of the lesion was maintained

Texture analysis was performed with the latest open-source 3D Slicer software (4.10.2-2019-05-30, available at https://www.slicer.org). CT images were extracted from the institution’s PACS data management system (PACSView, Medocs 3.00.123) in PACS format and transferred to 3D Slicer. The slice with the largest lesion diameter was selected. A 2D region of interest (ROI) was manually drawn by two radiologists, with 4 and 6 years of experience in abdominal radiology, respectively, in consensus (Fig. 2). The observers were blinded to histopathological grade. In difficult cases, when the lesion could not be clearly defined from non-enhanced images and to avoid incorrect inclusion of any adjacent vessels or tissues, additionally contrast-enhanced CT images also acquired for the CT-guided biopsy were used as a reference for placing 2D ROIs in the non-enhanced images.

3D Slicer software computed 372 features based on histogram analysis, co-occurrence matrix, and run-length matrix. Texture analysis was performed as a two-stage process. For image filtration purposes, Laplace of Gaussian (LoG) filters were applied, using sigma values of 0.5 (fine), 1.5 (medium), and 2.5 (coarse), in addition to the original CT image as previously advised [16–18]. Subsequently, quantitative texture analysis was performed. All texture features were extracted for each ROI.

### Statistical analysis

Differences in CTTA features between primary PDAC G2 and G3 tumors and between primary and metastatic PDAC were tested using the Mann–Whitney *U* test. For multiple testing a Bonferroni–Holm correction was performed in order to minimize Type I error. ROC analysis was performed to calculate sensitivities and specificities for differentiation between G2 and G3 tumors, whereas the Youden’s index was used for calculation of the best fit cut-off value. Statistical analysis was performed with commercially available software (IBM SPSS Statistics, Version 26, SPSS Inc.). A *p* value < 0.05 was considered statistically significant.

## Results

### Tumor size and density

The mean diameter of the ROIs in pancreatic lesions was 38 mm (range, 19–92 mm) with mean HU_tumor_ of 33 HU (range, 13–54 HU), whereas the mean size of the ROIs in liver lesions was 31 mm (range, 11–89 mm) with mean HU_tumor_ of 34 HU (range, 18–49 HU). No statistically significant differences in lesion size or mean density were detected between G2 and G3 tumors.

### CTTA of primary G2 versus G3 tumors

With the initially accepted significance level of *p* < 0.05, a total of 49 texture features were statistically significantly different between primary G2 and G3 tumors using Mann–Whitney *U*-Test. After Bonferroni–Holm correction for multiple testing, three texture features remained significantly different; ROC analysis of these features yielded AUCs between 0.766 and 0.866.

**Fig. 3:**
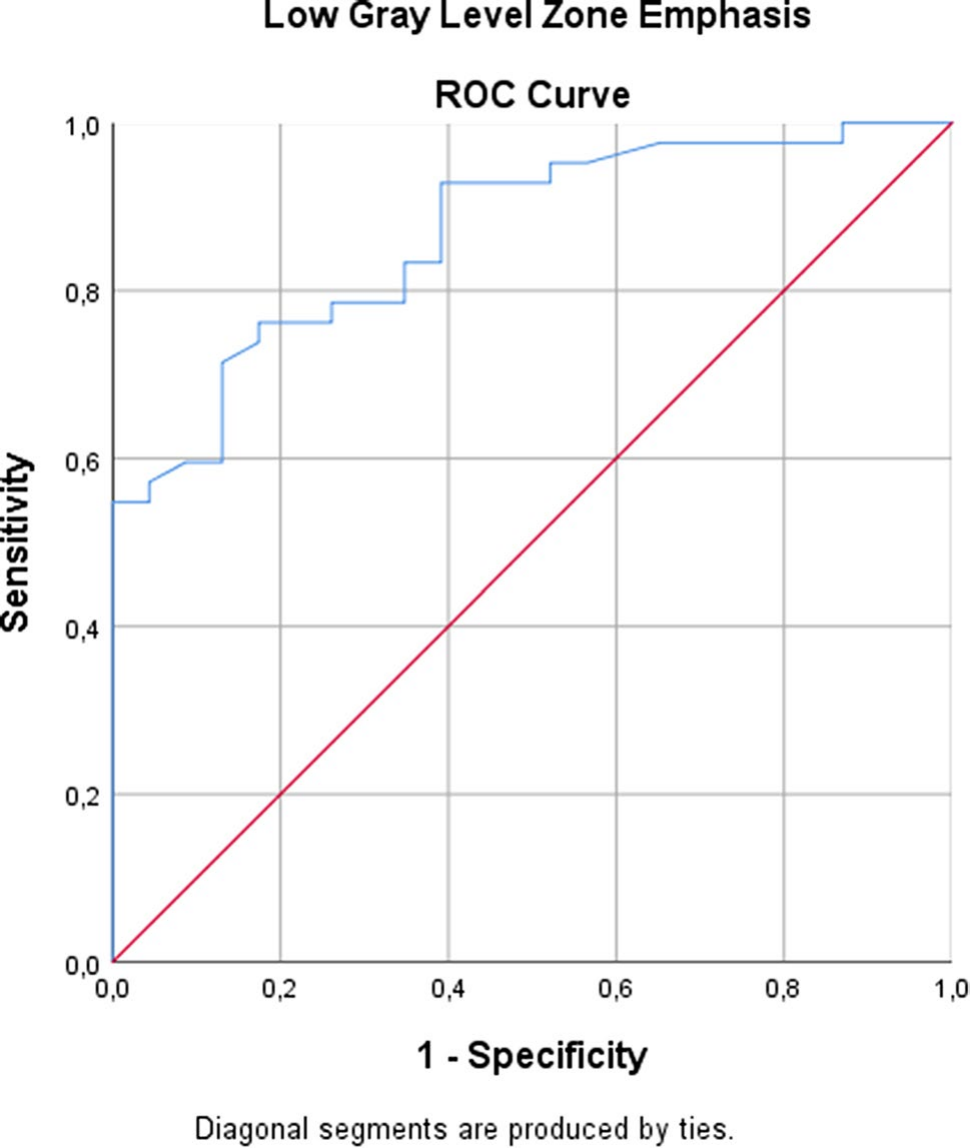
Receiver operating characteristic (ROC) curve for the feature “low gray-level zone emphasis (LoG 0)” giving an AUC value of 0.866 for the diagnosis of histopathological grade G3 in primary PDAC with a sensitivity and a specificity of 0.76 and 0.83, respectively, using 0.482 as a cut-off value

The feature “Low Gray-Level Zone Emphasis (LoG 0)” had the largest AUC of 0.866. Using a cut-off value of 0.482 this resulted in a sensitivity, a specificity, and Youden’s index of 0.76, 0.83, and 0.59, respectively (Fig. 3). On the other hand “Cluster shade” (AUC of 0.840) and “High Gray-Level Zone Emphasis” (AUC of 0.766) were also statistically significant. A detailed summary of the results is provided in Table 2.

**Table 2:** ROC analysis of texture features with statistically significant differences between primary G2 and G3 pancreatic adenocarcinomas after Bonferroni–Holm correction

Texture feature	Feature class	LoG filter (mm)	AUC	SD	CI 95%	*p* value
Low gray-level zone emphasis	GLSZM	0	0.866	0.04	0.781–0.952	< 0.001
Cluster shade	GLCM	0	0.840	0.05	0.734–0.945	< 0.001
High gray-level zone emphasis	GLSZM	0	0.766	0.06	0.651–0.880	< 0.001

*LoG* Laplace of Gaussian filter, *AUC* area under the curve, *SD* standard deviation, *CI* confidential interval 95%, *GLSZM* Gray-level size zone matrix features, *GLCM* Gray-level co-occurrence matrix features

“Low Gray-Level Zone Emphasis” and “High Gray-Level Zone Emphasis” are specific features of a Gray-Level Size Zone Matrix (GLSZM), which quantifies groups of voxels of the same gray level that are connected with each other. Cluster shade on the other hand is part of the Gray-Level Co-occurrence Matrix (GLCM) which describes certain combinations of gray-level intensity pairs within the region of interest [19].

### CTTA of primary PDAC versus hepatic metastasis

Mann–Whitney *U* test revealed 115 texture features that were significantly different between primary pancreatic carcinomas and hepatic metastases, applying the initial significance level of *p* < 0.05. After Bonferroni–Holm correction for multiple testing, 54 texture features remained statistically significant. ROC analysis of these features yielded AUCs between 0.710 and 0.879. The parameter “Low Gray-Level Emphasis (LoG 2.5)” showed the largest AUC (0.929). Low Gray-Level Emphasis belongs to the Gray-Level Dependence Matrix (GLDM), which quantifies voxels of a certain gray-level intensity in regard to a specific center voxel [19].

In total 3 first-order parameters were found statistically significant applying two different LoG filters (1.5, 2.5). Median as the median gray-level intensity found in the region of interest had the highest AUC of all first-order features. Several second- and higher-order parameters belonging to Gray-Level Co-occurrence Matrix, Gray-Level Run-Length Matrix, and Gray-Level Dependence Matrix were significantly different between primary PDAC and hepatic lesions when using LoG filters of 1.5 and 2.5. The most significant features with AUCs > 0.850 are listed in Table 3.

**Table 3:** Texture features that had an AUC ≥ 0.850 for differentiation between primary PDAC and hepatic PDAC metastasis

Texture feature	LoG filter (mm)	AUC	SD	CI 95%	*p* value
*First order*					
Median	2.5	0.907	0.028	0.853–0.962	< 0.00015
Median	1.5	0.872	0.033	0.807–0.937	< 0.00015
Mean	2.5	0.880	0.033	0.815–0.945	< 0.00015
Mean	1.5	0.834	0.038	0.759–0.909	< 0.00015
10 percentile	2.5	0.870	0.035	0.800–0.939	< 0.00015
*Gray-level co-occurrence matrix*					
Joint average	2.5	0.928	0.022	0.876–0.974	< 0.00015
Joint average	1.5	0.866	0.034	0.800–0.931	< 0.00015
Sum average	2.5	0.927	0.024	0.879–0.974	< 0.00015
Sum average	1.5	0.864	0.035	0.799–0.930	< 0.00015
Autocorrelation	2.5	0.924	0.025	0.876–0.972	< 0.00015
Autocorrelation	1.5	0.862	0.034	0.796–0.929	< 0.00015
Cluster shade	1.5	0.862	0.035	0.794–0.930	< 0.00015
*Gray-level run-length matrix*					
Low gray-level run emphasis	2.5	0.903	0.027	0.849–0.956	< 0.00015
High gray-level run emphasis	2.5	0.885	0.030	0.826–0.944	< 0.00015
*Gray-level dependence matrix*					
Low gray-level emphasis	2.5	0.929	0.024	0.882–0.975	< 0.00015
Low gray-level emphasis	1.5	0.873	0.033	0.809–0.937	< 0.00015
High gray-level emphasis	2.5	0.926	0.024	0.879–0.937	< 0.00015
High gray-level emphasis	1.5	0.870	0.033	0.805–0.935	< 0.00015
Large dependence high gray-level emphasis	2.5	0.911	0.027	0.857–0.965	< 0.00015

*LoG* Laplace of Gaussian filter, *AUC* area under the ROC curve, *SD* standard deviation, *CI* confidence interval 95%, *p* value after Bonferroni–Holm correction

## Discussion

Tumor grading is an important prognostic marker for long-term survival of patients with PDAC and may influence the treatment selection [4, 5, 20]. We investigated whether CTTA in non-enhanced CT images can be used to predict the histopathological grade of differentiation of PDAC. Our study provides evidence suggesting that non-contrast CTTA has potential as a tool for non-invasive tumor grade prediction, as we found that three features differed significantly between G2 and G3 tumors. In addition, we detected highly significant differences in texture features between primary PDAC and liver metastases from PDAC.

As CTTA has already shown promising results in the prediction of long-term survival, risk of potential relapse, and response to therapy for different tumor entities in several organs [21–26], ROC analysis of primary PDAC revealed that the feature “low gray-level zone emphasis” without utilizing a LoG filter had the largest AUC and the best cut-off value. Our results also confirm that the feature “Cluster Shade” performs well in distinguishing G2 from G3 tumors, as previously reported in a study on contrast-enhanced CTTA of PDAC [13]. Unlike that study, we used non-enhanced CT scans for CTTA to eliminate a possible source of error and to create a more standardized dataset. As besides other factors, such as radiation dose and CT reconstruction, variations in contrast-enhanced CT scans are considered a major risk factor for low reproducibility of radiomic features in pancreatic lesions, the quality of our data may overcome this limitation [15, 27]. A recent analysis revealed that only about 5% of all radiomic features met a defined threshold for reproducibility (specifically, a concordance correlation coefficient > 0.9) when contrast-enhanced CT scans with different scan parameters, injection rates, and protocols were used [15]. All patients included in our study were examined with the same CT scanner and identical scan parameters in order to gain a more standardized dataset. However, since in non-enhanced scans the pancreatic lesion was sometimes difficult to differentiate from normal pancreatic parenchyma due to small differences in density, we decided to rely on 2D segmentation on the slice with the largest lesion diameter to avoid the inclusion of normal parenchyma, calcifications, adjacent blood vessels, or parts of the intestine. Whether 2D segmentation in CTTA is superior to 3D segmentation or vice versa is controversial. On the one hand, 2D segmentation reduces the influence of motion artifacts and breathing artifacts and ensures that no adjacent structure is mistakenly included; on the other hand, 3D segmentation depicts the tumor in its entirety and may therefore more reliably reflect the tumor’s properties [28, 29]. Another finding of our study was the substantial difference in CTTA features between primary PDAC and hepatic metastasis of PDAC. This may be explained by genetic mutations of tumor cells in distant metastases, different levels of expression of growth factors, and different degrees of angiogenesis and micronecrosis, as has already been shown for other tumor entities [30, 31]. Potentially these differences may be responsible for the mixed response of tumor diseases to subsequent treatment [32, 33]. It must be emphasized that due to the differences in CTTA features between primary and metastatic PDAC, CTTA may not be appropriate for characterizing liver lesions in patients with PDAC. However, this should be further investigated in comparison to other tumor entities.

Several limitations of our study need to be considered. First, due to its retrospective design, selection bias cannot be ruled out and may have influenced the results. Second, since non-enhanced CT scans were used for CTTA, segmentation may have been prone to errors due to reduced lesion conspicuity. However, segmentation of contrast-enhanced CT scans is believed to have a high risk of low reproducibility due to contrast medium-based variability. Third, determining the grading from biopsy material carries the risk of sampling error, as the grading of pancreatic tumors is usually supposed to be determined by the surgical specimen. However, taking three punch cylinders in different angulations as applied at our institution for image-guided biopsies should reduce the probability of this error to a minimum. In general due to limited spatial resolution a small lesion size could potentially cause difficulties in a proper segmentation. As far as our study population is concerned all lesions were greater than 1 cm, therefore an adequate quality of segmentation could be preserved. On a further note, a large number of CTTA features were examined in a low number of patients, thus elevating the risk of obtaining false-positive results. However, we tried to prevent this effect with Bonferroni–Holm correction for multiple testing. Further prospective studies are necessary to validate the results on a large scale, concentrating on few relevant CTTA features.

## Conclusion

CTTA of PDAC identified differences in texture features between primary G2 and G3 tumors that may be used in future deep learning algorithms for non-invasive assessment of PDAC. Furthermore, differences found in numerous texture features between primary and hepatic metastatic PDAC imply that CTTA of a metastatic lesion may not be useful for drawing conclusions about the histopathological characteristics of a primary lesion or vice versa.

## Abbreviations

AUC: Area under the curve
CT: Computed tomography
CTTA: Computed tomography texture analysis
GLCM: Gray-level co-occurrence matrix
GLSZM: Gray-level size zone matrix
HU: Hounsfield Units
LoG: Laplacian of Gaussian
PACS: Picture archiving and communication system
PDAC: Pancreatic ductal adenocarcinoma
ROC: Receiver operating characteristic
ROI: Region of interest

## References

[1] Rawla P, Sunkara T, Gaduputi V (2019). Epidemiology of pancreatic cancer: global trends, etiology and risk factors. World J Oncol.

[2] Rochefort MM, Ankeny JS, Kadera BE, Donald GW, Isacoff W, Wainberg ZA, Hines OJ, Donahue TR, Reber HA, Tomlinson JS (2013). Impact of tumor grade on pancreatic cancer prognosis: validation of a novel TNMG staging system. Ann Surg Oncol.

[3] Haeberle L, Esposito I (2019). Pathology of pancreatic cancer. Transl Gastroenterol Hepatol.

[4] Macias N, Sayagues JM, Esteban C, Iglesias M, Gonzalez LM, Quinones-Sampedro J, Gutierrez ML, Corchete LA, Abad MM, Bengoechea O, Munoz-Bellvis L (2018). Histologic tumor grade and preoperative bilary drainage are the unique independent prognostic factors of survival in pancreatic ductal adenocarcinoma patients after pancreaticoduodenectomy. J Clin Gastroenterol.

[5] Kuhlmann KF, de Castro SM, Wesseling JG, ten Kate FJ, Offerhaus GJ, Busch OR, van Gulik TM, Obertop H, Gouma DJ (2004). Surgical treatment of pancreatic adenocarcinoma; actual survival and prognostic factors in 343 patients. Eur J Cancer.

[6] Nurmi A, Mustonen H, Parviainen H, Peltola K, Haglund C, Seppanen H (2018). Neoadjuvant therapy offers longer survival than upfront surgery for poorly differentiated and higher stage pancreatic cancer. Acta Oncol.

[7] Jamal-Hanjani M, Quezada SA, Larkin J, Swanton C (2015). Translational implications of tumor heterogeneity. Clin Cancer Res.

[8] Jamal-Hanjani M, Wilson GA, McGranahan N, Birkbak NJ, Watkins TBK, Veeriah S, Shafi S, Johnson DH, Mitter R, Rosenthal R, Salm M, Horswell S, Escudero M, Matthews N, Rowan A, Chambers T, Moore DA, Turajlic S, Xu H, Lee SM, Forster MD, Ahmad T, Hiley CT, Abbosh C, Falzon M, Borg E, Marafioti T, Lawrence D, Hayward M, Kolvekar S, Panagiotopoulos N, Janes SM, Thakrar R, Ahmed A, Blackhall F, Summers Y, Shah R, Joseph L, Quinn AM, Crosbie PA, Naidu B, Middleton G, Langman G, Trotter S, Nicolson M, Remmen H, Kerr K, Chetty M, Gomersall L, Fennell DA, Nakas A, Rathinam S, Anand G, Khan S, Russell P, Ezhil V, Ismail B, Irvin-Sellers M, Prakash V, Lester JF, Kornaszewska M, Attanoos R, Adams H, Davies H, Dentro S, Taniere P, O'Sullivan B, Lowe HL, Hartley JA, Iles N, Bell H, Ngai Y, Shaw JA, Herrero J, Szallasi Z, Schwarz RF, Stewart A, Quezada SA, Le Quesne J, Van Loo P, Dive C, Hackshaw A, Swanton C, T.R. Consortium (2017). Tracking the evolution of non-small-cell lung cancer. N Engl J Med.

[9] Marusyk A, Polyak K (2010). Tumor heterogeneity: causes and consequences. Biochim Biophys Acta.

[10] Oh BY, Shin HT, Yun JW, Kim KT, Kim J, Bae JS, Cho YB, Lee WY, Yun SH, Park YA, Park YH, Im YH, Lee J, Joung JG, Kim HC, Park WY (2019). Intratumor heterogeneity inferred from targeted deep sequencing as a prognostic indicator. Sci Rep.

[11] Yun G, Kim YH, Lee YJ, Kim B, Hwang JH, Choi DJ (2018). Tumor heterogeneity of pancreas head cancer assessed by CT texture analysis: association with survival outcomes after curative resection. Sci Rep.

[12] Sandrasegaran K, Lin Y, Asare-Sawiri M, Taiyini T, Tann M (2019). CT texture analysis of pancreatic cancer. Eur Radiol.

[13] Qiu W, Duan N, Chen X, Ren S, Zhang Y, Wang Z, Chen R (2019). Pancreatic ductal adenocarcinoma: machine learning-based quantitative computed tomography texture analysis for prediction of histopathological grade. Cancer Manag Res.

[14] Larue R, van Timmeren JE, de Jong EEC, Feliciani G, Leijenaar RTH, Schreurs WMJ, Sosef MN, Raat F, van der Zande FHR, Das M, van Elmpt W, Lambin P (2017). Influence of gray level discretization on radiomic feature stability for different CT scanners, tube currents and slice thicknesses: a comprehensive phantom study. Acta Oncol.

[15] Yamashita R, Perrin T, Chakraborty J, Chou JF, Horvat N, Koszalka MA, Midya A, Gonen M, Allen P, Jarnagin WR, Simpson AL, Do RKG (2020). Radiomic feature reproducibility in contrast-enhanced CT of the pancreas is affected by variabilities in scan parameters and manual segmentation. Eur Radiol.

[16] Ganeshan B, Abaleke S, Young RC, Chatwin CR, Miles KA (2010). Texture analysis of non-small cell lung cancer on unenhanced computed tomography: initial evidence for a relationship with tumour glucose metabolism and stage. Cancer Imaging.

[17] Ganeshan B, Miles KA, Young RC, Chatwin CR (2009). Texture analysis in non-contrast enhanced CT: impact of malignancy on texture in apparently disease-free areas of the liver. Eur J Radiol.

[18] Yip C, Landau D, Kozarski R, Ganeshan B, Thomas R, Michaelidou A, Goh V (2014). Primary esophageal cancer: heterogeneity as potential prognostic biomarker in patients treated with definitive chemotherapy and radiation therapy. Radiology.

[19] van Griethuysen JJM, Fedorov A, Parmar C, Hosny A, Aucoin N, Narayan V, Beets-Tan RGH, Fillion-Robin JC, Pieper S, Aerts H (2017). Computational radiomics system to decode the radiographic phenotype. Cancer Res.

[20] Liu L, Xu HX, He M, Wang W, Wang WQ, Wu CT, Wei RQ, Liang Y, Gao HL, Liu C, Xu J, Long J, Ni QX, Shao CH, Wang J, Yu XJ (2018). A novel scoring system predicts postsurgical survival and adjuvant chemotherapeutic benefits in patients with pancreatic adenocarcinoma: implications for AJCC-TNM staging. Surgery.

[21] Ahn SY, Park CM, Park SJ, Kim HJ, Song C, Lee SM, McAdams HP, Goo JM (2015). Prognostic value of computed tomography texture features in non-small cell lung cancers treated with definitive concomitant chemoradiotherapy. Invest Radiol.

[22] Bayanati H, Thornhill RE, Souza CA, Sethi-Virmani V, Gupta A, Maziak D, Amjadi K, Dennie C (2015). Quantitative CT texture and shape analysis: can it differentiate benign and malignant mediastinal lymph nodes in patients with primary lung cancer?. Eur Radiol.

[23] Ganeshan B, Skogen K, Pressney I, Coutroubis D, Miles K (2012). Tumour heterogeneity in oesophageal cancer assessed by CT texture analysis: preliminary evidence of an association with tumour metabolism, stage, and survival. Clin Radiol.

[24] Goh V, Ganeshan B, Nathan P, Juttla JK, Vinayan A, Miles KA (2011). Assessment of response to tyrosine kinase inhibitors in metastatic renal cell cancer: CT texture as a predictive biomarker. Radiology.

[25] Tian F, Hayano K, Kambadakone AR, Sahani DV (2015). Response assessment to neoadjuvant therapy in soft tissue sarcomas: using CT texture analysis in comparison to tumor size, density, and perfusion. Abdom Imaging.

[26] Zhang H, Graham CM, Elci O, Griswold ME, Zhang X, Khan MA, Pitman K, Caudell JJ, Hamilton RD, Ganeshan B, Smith AD (2013). Locally advanced squamous cell carcinoma of the head and neck: CT texture and histogram analysis allow independent prediction of overall survival in patients treated with induction chemotherapy. Radiology.

[27] Meyer M, Ronald J, Vernuccio F, Nelson RC, Ramirez-Giraldo JC, Solomon J, Patel BN, Samei E, Marin D (2019). Reproducibility of CT radiomic features within the same patient: influence of radiation dose and CT reconstruction settings. Radiology.

[28] Ng F, Kozarski R, Ganeshan B, Goh V (2013). Assessment of tumor heterogeneity by CT texture analysis: can the largest cross-sectional area be used as an alternative to whole tumor analysis?. Eur J Radiol.

[29] Shen C, Liu Z, Guan M, Song J, Lian Y, Wang S, Tang Z, Dong D, Kong L, Wang M, Shi D, Tian J (2017). 2D and 3D CT radiomics features prognostic performance comparison in non-small cell lung cancer. Transl Oncol.

[30] Baisse B, Bouzourene H, Saraga EP, Bosman FT, Benhattar J (2001). Intratumor genetic heterogeneity in advanced human colorectal adenocarcinoma. Int J Cancer.

[31] Losi L, Baisse B, Bouzourene H, Benhattar J (2005). Evolution of intratumoral genetic heterogeneity during colorectal cancer progression. Carcinogenesis.

[32] Dong ZY, Zhai HR, Hou QY, Su J, Liu SY, Yan HH, Li YS, Chen ZY, Zhong WZ, Wu YL (2017). Mixed responses to systemic therapy revealed potential genetic heterogeneity and poor survival in patients with non-small cell lung cancer. Oncologist.

[33] Wu JB, Sarmiento AL, Fiset PO, Lazaris A, Metrakos P, Petrillo S, Gao ZH (2019). Histologic features and genomic alterations of primary colorectal adenocarcinoma predict growth patterns of liver metastasis. World J Gastroenterol.

